# Reduced pre-movement subthalamic beta desynchronization marks motor deficit in Parkinson’s disease

**DOI:** 10.1093/braincomms/fcag245

**Published:** 2026-06-25

**Authors:** Gang Seo, Kevin B Wilkins, Helen M Bronte-Stewart

**Affiliations:** Department of Neurology and Neurological Sciences, Stanford University School of Medicine, Stanford, CA 94305, USA; Department of Neurology and Neurological Sciences, Stanford University School of Medicine, Stanford, CA 94305, USA; Department of Neurology and Neurological Sciences, Stanford University School of Medicine, Stanford, CA 94305, USA; Department of Neurosurgery, Stanford University School of Medicine, Stanford, CA 94305, USA

**Keywords:** electrophysiology, motor control, movement disorder, intracranial recording, parkinsonian

## Abstract

Abnormal beta-band (13–30 Hz) oscillations in the subthalamic nucleus are a well-established biomarker of motor dysfunction in Parkinson's disease. While most prior work has focused on beta activity during rest or sustained movement, far less is known about its transient dynamics during movement preparation—a critical phase in which suppression of beta-band activity (beta desynchronization) is thought to facilitate motor circuit readiness. Here, we delineate subthalamic beta-band activity specifically in the pre-movement initiation window across diverse motor tasks and demonstrate its association with both clinical motor impairment severity and subsequent movement acceleration. We recorded subthalamic nucleus local field potentials and kinematics in sixteen individuals with Parkinson's disease (10 males, 6 females; mean age 57.9 ± 10.5 years) implanted with sensing deep brain stimulation systems (Medtronic Activa® PC+S). While off medication and stimulation, participants performed cued motor tasks, including sit-to-stand, stand-to-walk and wrist flexion-extension. Pre-movement beta desynchronization, quantified as beta power normalized to resting baseline, was extracted and analysed using linear mixed-effects models to determine its relationship with clinical impairment severity and movement acceleration. Across all tasks, we observed robust pre-movement beta desynchronization in the subthalamic nucleus (*P* < 0.001). Critically, reduced desynchronization was associated with greater motor impairment, particularly bradykinesia (*P* < 0.001). This association appeared stronger during the more complex stand-to-walk task and was linked to reduced movement acceleration, as measured by acceleration indices (*P* < 0.05). A strong link between greater pre-movement beta desynchronization, less severe bradykinesia and more vigorous movement suggests that impaired beta modulation reflects disruptions in motor planning, delayed recruitment of motor networks and excessive basal ganglia inhibition. These circuit-level abnormalities likely contribute to the difficulties individuals with Parkinson's disease face in initiating and executing movements, offering valuable insight into the neurophysiological basis of motor dysfunction in Parkinson's disease.

## Teaser

This work links a specific preparatory brain signal to motor deficit in Parkinson’s disease. Seo *et al*. found that reduced suppression of beta brainwaves in the subthalamic nucleus just before a movement reflects motor impairment, offering valuable insight into the neural basis of motor dysfunction in Parkinson's disease.

## Graphical Abstract

**Graphical Abstract:**
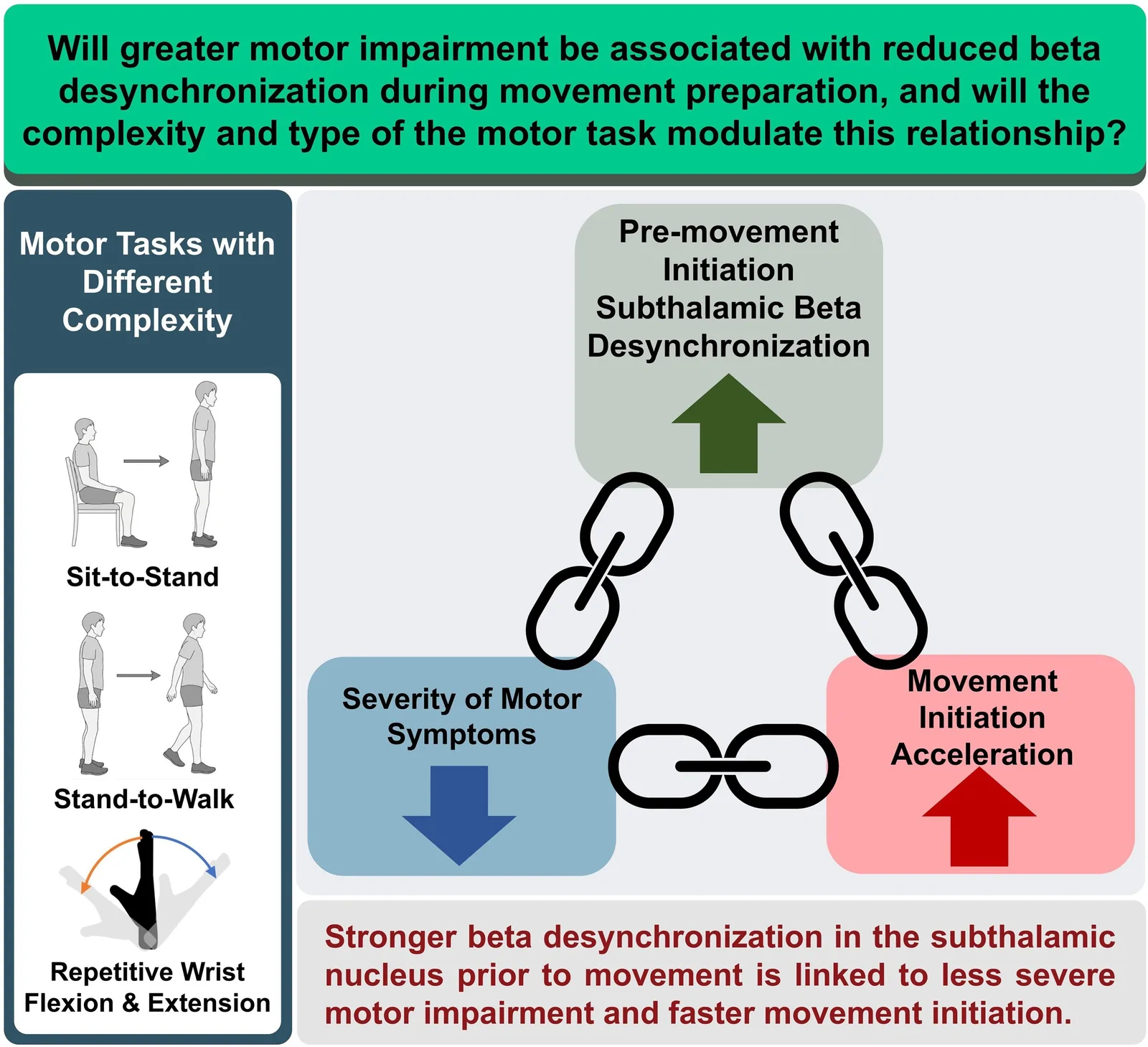

## Introduction

Parkinson’s disease is a neurodegenerative disorder characterized by cardinal motor symptoms including rest tremor, bradykinesia, rigidity and postural instability, significantly impairing patients’ daily functioning and quality of life.^1,2^ Additional motor manifestations, such as gait disturbances, freezing of gait and impaired balance, further complicate clinical management and substantially contribute to disability and reduced independence.^3-5^ These motor symptoms primarily result from dysfunction within basal ganglia-thalamo-cortical circuits, largely due to degeneration of dopaminergic neurons in the substantia nigra pars compacta.^6,7^

Accumulating evidence from electrophysiological studies in Parkinson’s disease patients has identified abnormal oscillatory activity in the beta frequency range (13–30 Hz) within the basal ganglia, particularly the subthalamic nucleus (STN), as a robust biomarker of motor impairment.^8-10^ Excessive synchronization in the beta frequency range is closely linked to bradykinesia and rigidity, with numerous studies demonstrating that elevated beta power correlates with symptom severity.^11-16^ Therapeutic interventions such as dopaminergic medication and deep brain stimulation (DBS) attenuate beta activity, correlating with clinical improvement.^9,16-19^ These findings underscore the clinical relevance of beta oscillations and support their role as both a marker and a potential therapeutic target in Parkinson’s disease.^10,20^

These insights have led to a focus on the transient neural dynamics during the pre-movement or movement preparation phase. Foundational work has established that beta desynchronization (a decrease in beta-band power) in the STN occurs prior to externally cued movements^21,22^ and that its latency correlates with reaction time. Crucially, this pre-movement modulation has also been linked to dopamine state and clinical motor impairment. For instance, Doyle *et al*.^23^ demonstrated a correlation between the extent of event-related desynchronization and Unified Parkinson’s Disease Rating Scale part III (UPDRS III) motor scores, showing that this relationship is modulated by levodopa.

While this prior work established a key link using simple, self-paced upper-extremity tasks, our study aims to build upon these findings. We seek to characterize the magnitude of pre-movement beta desynchronization and correlate it with motor impairment severity across a battery of more complex, functionally relevant, whole-body functional tasks (e.g. sit-to-stand, stand-to-walk). Furthermore, we extend this investigation by using objective, quantitative kinematics to measure subsequent movement acceleration, rather than relying solely on clinical scores. Addressing these questions is essential for a more comprehensive understanding of how subcortical beta-band dynamics relate to functional motor impairment in Parkinson’s disease and may have important implications for the development of biomarkers and individualized therapeutic strategies.^20,24^ We hypothesize that greater motor impairment will be associated with reduced beta desynchronization during movement preparation, and that the complexity and type of the motor task will modulate this relationship.

To test these hypotheses, we enrolled sixteen individuals with Parkinson’s disease implanted with a sensing DBS system. Participants performed a battery of externally cued motor tasks—including sit-to-stand, stand-to-walk and repetitive wrist flexion-extension (rWFE)—while simultaneous recordings of STN local field potentials (LFPs) and kinematic data were obtained in the medication- and stimulation-off state. This approach enables a detailed investigation of the neural correlates of movement preparation and their association with clinical and kinematic measures of motor impairment in Parkinson’s disease, thereby providing novel insights into the functional role of subcortical beta activity in motor preparation and impairment in Parkinson’s disease.

## Materials and methods

### Participants

**Figure 1:**
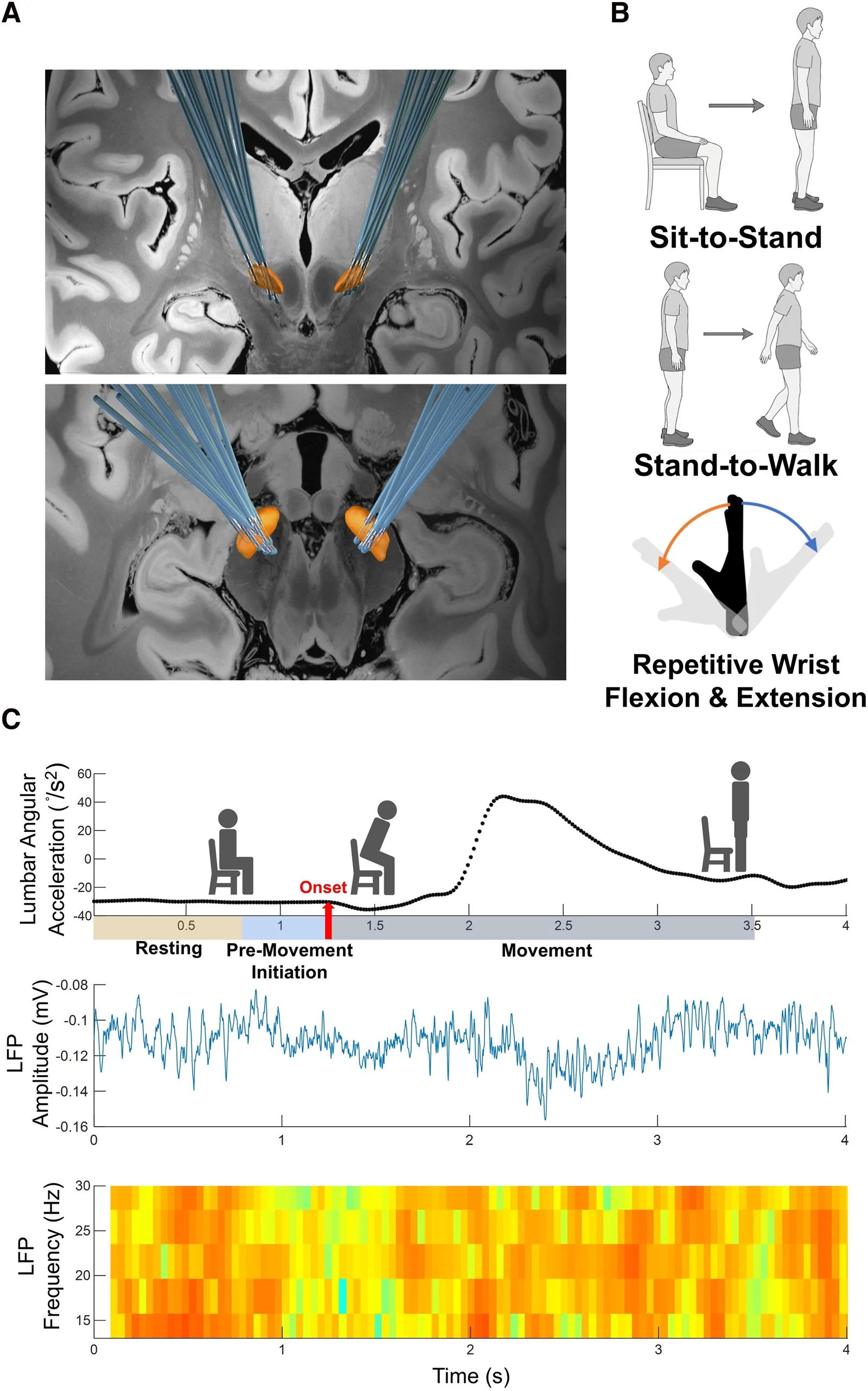
**Subthalamic nucleus (STN) deep brain stimulation (DBS) lead localization and overview of motor tasks and experimental design.** (**A**) Coronal (*top*) and axial (*bottom*) view of STN DBS lead localization (*n* = 16). (**B**) Motor tasks (from *top* to *bottom*: sit-to-stand, stand-to-walk and repetitive wrist flexion and extension). (**C**) Example data from the sit-to-stand task: time-synchronized angular acceleration of the lumbar region (*top*), raw STN local field potential (LFP) signal (*middle*) and spectrogram of beta-band STN LFP activity (*bottom*) during different phases of the sit-to-stand task.

Sixteen individuals (10 males) with clinically established Parkinson’s disease participated in this study within 12 months following DBS implantation. All participants underwent implantation of an investigational sensing neurostimulator (Medtronic Activa® PC+S) with bilateral DBS leads (Medtronic model 3389) targeting the sensorimotor region of the STN (Fig. 1A, Supplementary Table 1). Preoperative selection criteria and surgical methods have been described in our previous study.^25^ All study participants provided written informed consent in accordance with the Declaration of Helsinki prior to participation. The study protocol was approved by the U.S. Food and Drug Administration (Investigational Device Exemption) and the Stanford University Institutional Review Board.

### Experimental protocol

All experimental procedures were conducted after a standardized medication washout period: long-acting dopamine agonists were discontinued at least 48 h prior, dopamine agonists and controlled-release carbidopa/levodopa at least 24 h prior and short-acting medications at least 12 h prior to testing. Additionally, STN DBS was turned off for 60–75 min before the rWFE task and 15–60 min before the sit-to-stand and stand-to-walk tasks, durations previously demonstrated to be sufficient for washout of DBS effects on STN LFPs and motor symptoms.^19,26-28^

The participants performed three motor tasks—sit-to-stand, stand-to-walk and seated rWFE—during assessment visits and the number of visits varied across participants and tasks. At each visit, one to three trials of each task were performed, and the Movement Disorder Society-Unified Parkinson’s Disease Rating Scale part III (MDS-UPDRS III) was assessed.

In the sit-to-stand task, they began in a seated resting position and stood up at their own pace following a verbal cue. The stand-to-walk task required participants to start from a standing resting position and initiate walking at their own pace after receiving a verbal cue. Lastly, during the rWFE task, participants started from a seated resting position and, upon a verbal cue, performed rapid wrist flexion-extension movements continuously for 30 s. The rWFE task was performed separately with each arm, in randomized order (Fig. 1B).

### Data acquisition and analysis

#### Kinematic data

Kinematic data were collected using inertial measurement unit (IMU) sensors. For the sit-to-stand and stand-to-walk tasks, triaxial IMU sensors (Opals and Mobility Lab) were placed on the lower back (lumbar region) and both shanks. For the rWFE task, uniaxial wrist IMU sensors (Motus Bioengineering, Inc.) were attached to the dorsum of each hand. Angular acceleration data from lumbar and shank sensors were recorded at 128 Hz, while angular velocity data from wrist sensors were recorded at 1 kHz.

Both angular acceleration and angular velocity data were baseline-corrected by subtracting the mean value of the resting phase and subsequently low-pass filtered using a zero-lag fourth-order Butterworth filter with a cut-off frequency of 4 Hz.^15,16,29^ To identify movement onset for the sit-to-stand and stand-to-walk tasks, the vector magnitude (VM) of triaxial angular acceleration (*A_x_, A_y_, A_z_*) was calculated as


(1) $$VM=\sqrt{A_{x}{\;}^{2}+A_{y}{\;}^{2}+A_{z}{\;}^{2}}.$$

For the rWFE task, filtered uniaxial angular velocity data were used to detect movement onset. Movement onset was defined as the time point at which VM or angular velocity exceeded a threshold of four standard deviations above the mean amplitude of the resting phase. In cases where severe resting noise precluded threshold-based detection (∼8% of trials), onset was defined using a semi-automated back-search algorithm to minimize observer bias. The operator identified the initial movement peak, and the algorithm searched backward to locate the onset where the signal slope (calculated over five consecutive points) decreased by at least 70%. For sit-to-stand and stand-to-walk tasks, lumbar angular acceleration was primarily used for movement onset detection; however, shank angular acceleration was used if it indicated an earlier onset (Fig. 1C).

To characterize movement vigour immediately following movement initiation, an acceleration index (*AI*) was calculated for each trial of the sit-to-stand and stand-to-walk tasks as


(2) $$AI=\;\frac{VM_{pk2pk}}{T_{mov}},$$

where *T*_mov_ is the duration of the movement and *VM_pk2pk_* is the peak-to-peak amplitude of VM during *T*_mov_. For sit-to-stand, *T*_mov_ was defined as the interval from the seated position to complete standing and *VM_pk2pk_* of lumbar acceleration was used; for stand-to-walk, *T*_mov_ was defined as the interval from standing rest to completion of the first step and *VM_pk2pk_* of shank acceleration was used. Trials in which participants failed to execute a distinct initial step were excluded from the analysis.

#### Neural data

For STN LFP recording, sense-friendly configurations of the Activa® PC+S system (electrode contact pairs on the DBS leads: 0–2 or 1–3 for left STN and 8–10 or 9–11 for right STN; mean ± STD of impedance: 4.5 ± 1.2 kΩ) were used (Supplementary Table 2). The contact pair exhibiting the highest beta power was selected during an initial programming visit after implantation and remained consistent across future experimental visits. LFP signals from each STN were sampled at 422 Hz and band-pass filtered between 0.5 and 100 Hz within the device.^30^

Neural data analyses were conducted offline using MATLAB (MathWorks, Natick, MA). Power spectral density was estimated using Welch’s method with a 0.25-s sliding Hanning window and 50% overlap. (This window size was selected to assume quasi-stationarity while retaining sufficient temporal resolution to capture the brief pMI phase.) Beta-band (13–30 Hz) power spectral density was calculated for two distinct phases: the pre-movement initiation (pMI) phase (0.5 s preceding movement onset,^23^ selected to isolate the late preparatory phase of movement execution) and a stable resting phase (from 10 to 5 s before movement onset, selected to strictly define the baseline well before the verbal cue was administered to ensure freedom from contamination by cue-related processing or immediate anticipatory activity).

For each trial, pMI beta power values were normalized to their corresponding resting phase beta power, converted to a logarithmic scale (dB) and averaged across trials for each visit per participant. Given that the pMI beta power was normalized to the resting phase beta power, the normalized value indicates the degree of beta desynchronization in this study. To compare resting beta power across tasks, resting phase beta power values were log-transformed and *Z*-score normalized for each trial, then averaged across trials for each visit per participant.

#### Synchronization of kinematic and neural data

Kinematic and neural data were synchronized using a Power1401 data acquisition interface and Spike software (version 2.7, Cambridge Electronic Design, Ltd., Cambridge, England). To prevent signal contamination, synchronization was performed during a dedicated preparatory session prior to the motor tasks. To achieve synchronization between neural and kinematic recordings, brief neurostimulation pulses (20 Hz, 1.5 V) were delivered through one of the DBS leads. These stimulation pulses produced artifacts detectable by both the implanted DBS sensing system and the external Spike recording system. The Spike system recorded these artifacts via surface EEG electrodes placed on the forehead, collarbone and skin above the implanted neurostimulator, sampled at 1 kHz. Based on the sampling rates of the DBS (422 Hz) and external (1 kHz) systems, the maximum theoretical timing jitter for this alignment is estimated at <5 ms. Motus IMU data were simultaneously acquired along with signals from the surface EEG electrodes. Additionally, the triaxial IMU system delivered a transistor-transistor logic pulse to the Spike system at the start of recording, which was captured concurrently with the EEG signals. Following data collection, neural and kinematic data files were co-registered offline using MATLAB.

### Statistical analysis

Statistical analyses were performed using MATLAB, with statistical significance set at *α* = 0.05. To confirm significant beta desynchronization during the pMI phase, pMI beta power values divided by resting beta power were first averaged across trials for each participant separately for each task. Subsequently, Wilcoxon signed-rank tests were conducted on these participant-level averages for each task to determine whether the median normalized beta power was significantly lower than zero.

Differences in resting beta power and pMI beta desynchronization across tasks were assessed using linear mixed-effects models (LMEM). These models included task as a fixed effect and participant as a random effect (with random intercepts and slopes), explicitly accounting for the nesting of hemispheres within subjects and unequal observations per participant. Pairwise comparisons between tasks were conducted using linear contrasts of fixed-effect coefficients, with Bonferroni corrections applied for multiple comparisons.

To examine the relationship between pMI beta desynchronization and the overall severity of clinical motor impairment, LMEM analyses were performed separately for each task. Each model included the degree of pMI beta desynchronization (normalized pMI beta power) as the fixed-effect predictor, MDS-UPDRS III total scores as the dependent variable, and subject-specific random intercepts and slopes to account for repeated measures within subjects. The strength of these associations was quantified using the beta coefficient (β) of the fixed-effect predictor, along with corresponding 95% confidence intervals, *t*-values and *P*-values.

Further analyses explored specific clinical relationships by repeating LMEM analyses using MDS-UPDRS III subscores as dependent variables. The subscores analysed were bradykinesia (sum of sections 3.4–3.8), rigidity (section 3.3), tremor (sections 3.15–3.18) and axial symptoms (sections 3.9, 3.10, 3.12–3.14). Separate models were constructed for each subscore and task, again including subject-specific random intercepts and slopes.

Pairwise linear contrasts of fixed-effect coefficients were then conducted to statistically compare the strength of associations (slopes) between tasks or between subscores within each task. Bonferroni corrections were applied to control for multiple comparisons.

For the rWFE task, analyses were conducted separately for the more-affected (MA) and less-affected (LA) sides, as well as combined across both sides, following the same LMEM and pairwise contrast procedures described above.

Finally, LMEM analyses were performed to examine relationships between pMI beta desynchronization and movement kinematics, using the AI as the dependent variable, the degree of pMI beta desynchronization (normalized pMI beta power) as the fixed-effect predictor, and subject-specific random intercepts and slopes. Similar LMEM analyses were also conducted to assess the relationship between AI and MDS-UPDRS III subscores.

## Results

This study included 16 participants (10 males) with a mean age of 57.9 ± 10.5 years and a mean disease duration of 8.3 ± 3.4 years. The mean number of visits for the sit-to-stand, stand-to-walk and rWFE tasks was 2.4 ± 1.5, 2.2 ± 1.5 and 3.6 ± 1.3, respectively. Across these visits, the mean pre-operative, off-medication MDS-UPDRS III score was 46.4 ± 9.3 (Table 1).

**Table 1:** Participant demographics

ID	Age	Sex	Disease duration (years)	Pre-op UPDRS III (off meds)	Number of visits (sit-to-stand)	Number of visits (stand-to-walk)	Number of visits (rWFE)
1	58.1	M	3.3	39	2	2	4
2	42.1	M	4.4	58	2	2	5
3	68.9	M	10.5	41	1	1	3
4	53.4	M	7.5	52	3	3	5
5	73.1	F	8.7	37	1	1	0
6	72.2	M	8.8	47	2	2	4
7	58.6	F	9.9	33	0	0	5
8	54.4	M	10.9	56	2	2	3
9	34.2	M	1.9	59	0	0	3
10	56.9	M	5.8	62	5	5	3
11	67.5	F	6.0	44	5	4	3
12	50.5	F	9.1	50	5	5	5
13	61.8	M	14.2	42	3	3	4
14	51.9	F	12.4	34	3	2	2
15	65.9	M	7.5	51	2	1	4
16	56.7	F	11.5	38	3	2	5
Avg ± SD	57.9 ± 10.5	10 M, 6 F	8.3 ± 3.4	46.4 ± 9.3	2.4 ± 1.5	2.2 ± 1.5	3.6 ± 1.3

### Consistent subthalamic beta desynchronization across different motor tasks

**Figure 2:**
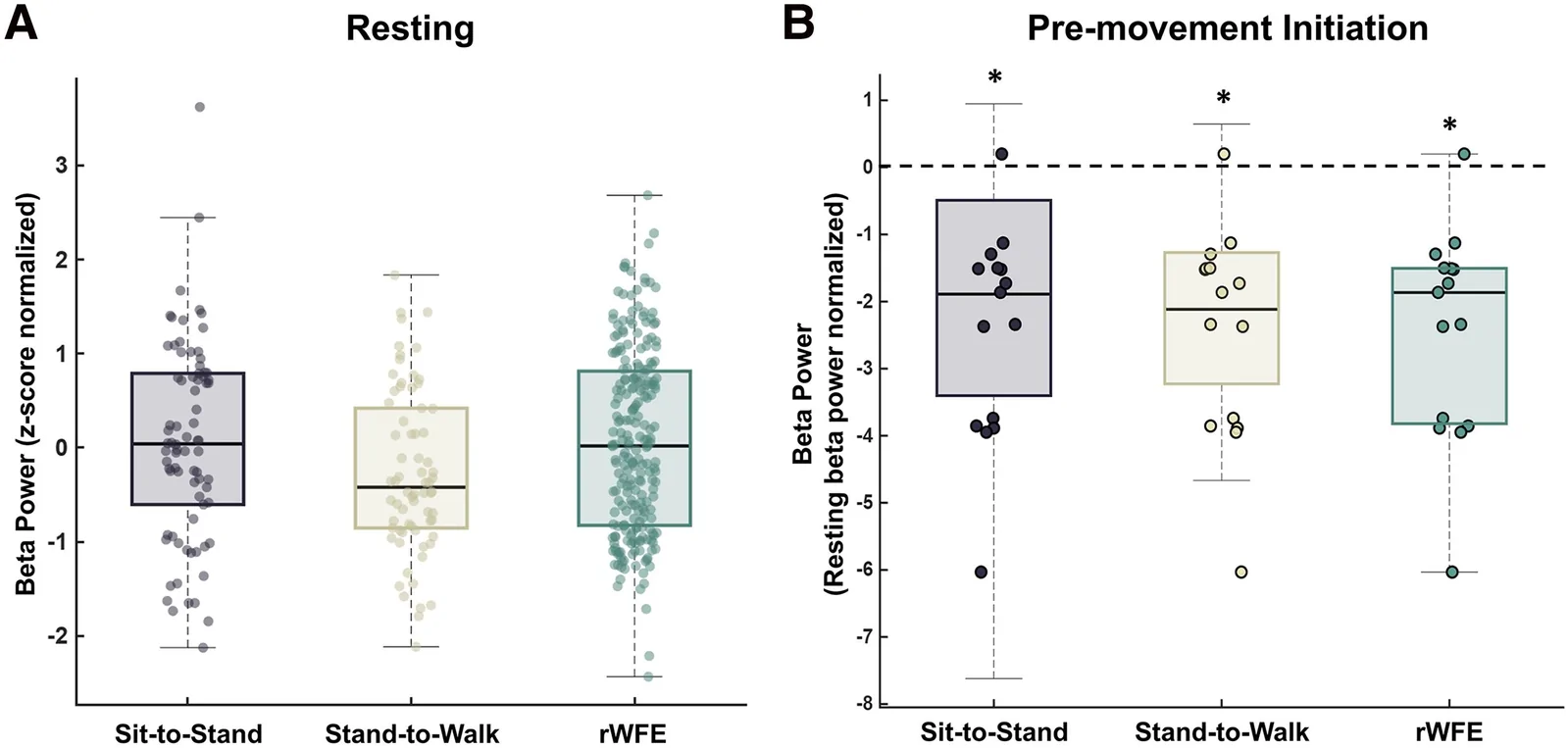
**Normalized subthalamic nucleus beta power for each motor task at different phases.** (**A**) *Z*-score normalized beta power during the resting phase. Each dot represents a single measurement per hemisphere, participant and visit. Beta power recorded during the repetitive wrist flexion and extension (rWFE) task was included for both the right and left wrist conditions (sit-to-stand, *n* = 78; stand-to-walk, *n* = 70; rWFE, *n* = 228; linear mixed-effects model; sit-to-stand and stand-to-walk, *β* = −0.33 [−0.64, −0.014], *t* = −2.1, *P* = 0.12; sit-to-stand and rWFE, *β* = −0.0020 [−0.25, 0.25], *t* = −0.010, *P* = 1.0; stand-to-walk and rWFE, *β* = −0.33 [−0.73, 0.075], *t* = −1.6, *P* = 0.053). (**B**) Degree of pre-movement initiation beta desynchronization (beta power during pre-movement initiation phase normalized by resting phase beta power). The dashed horizontal line indicates a normalized beta power of zero (no change from resting beta power). The outlined marker indicates the participant-level average beta power. Asterisks (*) indicate significant participant-level average beta desynchronization relative to zero (Wilcoxon signed-rank tests; sit-to-stand, *n* = 14, median = −1.9, *P* < 0.001; stand-to-walk, *n* = 14, median = −2.1, *P* < 0.001; rWFE, *n* = 15, median = −1.9, *P* < 0.001).

Normalized beta power in the STN did not significantly differ across the three motor tasks during either the resting or pMI phases. During the resting phase, normalized beta power was 0.060 ± 1.1 for sit-to-stand, −0.27 ± 0.87 for stand-to-walk and 0.060 ± 0.98 for rWFE. Although resting beta power appeared numerically lower during the stand-to-walk task compared to the other two tasks, this difference did not reach statistical significance (LMEM; sit-to-stand and stand-to-walk, *β* = −0.33 [−0.64, −0.014], *t* = −2.1, *P* = 0.12; sit-to-stand and rWFE, *β* = −0.0020 [−0.25, 0.25], *t* = −0.010, *P* = 1.0; stand-to-walk and rWFE, *β* = −0.33 [−0.73, 0.075], *t* = −1.6, *P* = 0.053; Fig. 2A).

During the pMI phase, participants consistently exhibited significant beta desynchronization (normalized beta power significantly lower than zero) across all tasks (Wilcoxon signed-rank tests; sit-to-stand, median = −1.9, *P* < 0.001; stand-to-walk, median = −2.1, *P* < 0.001; rWFE, median = −1.9, *P* < 0.001; Fig. 2B), indicating robust reductions in beta power relative to resting baseline. However, normalized beta power during the pMI phase did not significantly differ among the three tasks (LMEM; sit-to-stand and stand-to-walk, *β* = 0.32 [−0.48, 1.1], *t* = 0.79, *P* = 1.0; sit-to-stand and rWFE, *β* = 0.04 [−0.61, 0.70], *t* = 0.13, *P* = 1.0; stand-to-walk and rWFE, *β* = 0.04 [−0.61, 0.70], *t* = 0.13, *P* = 1.0; Fig. 2B).

### Association between pre-movement subthalamic beta desynchronization and severity of motor impairment

**Figure 3:**
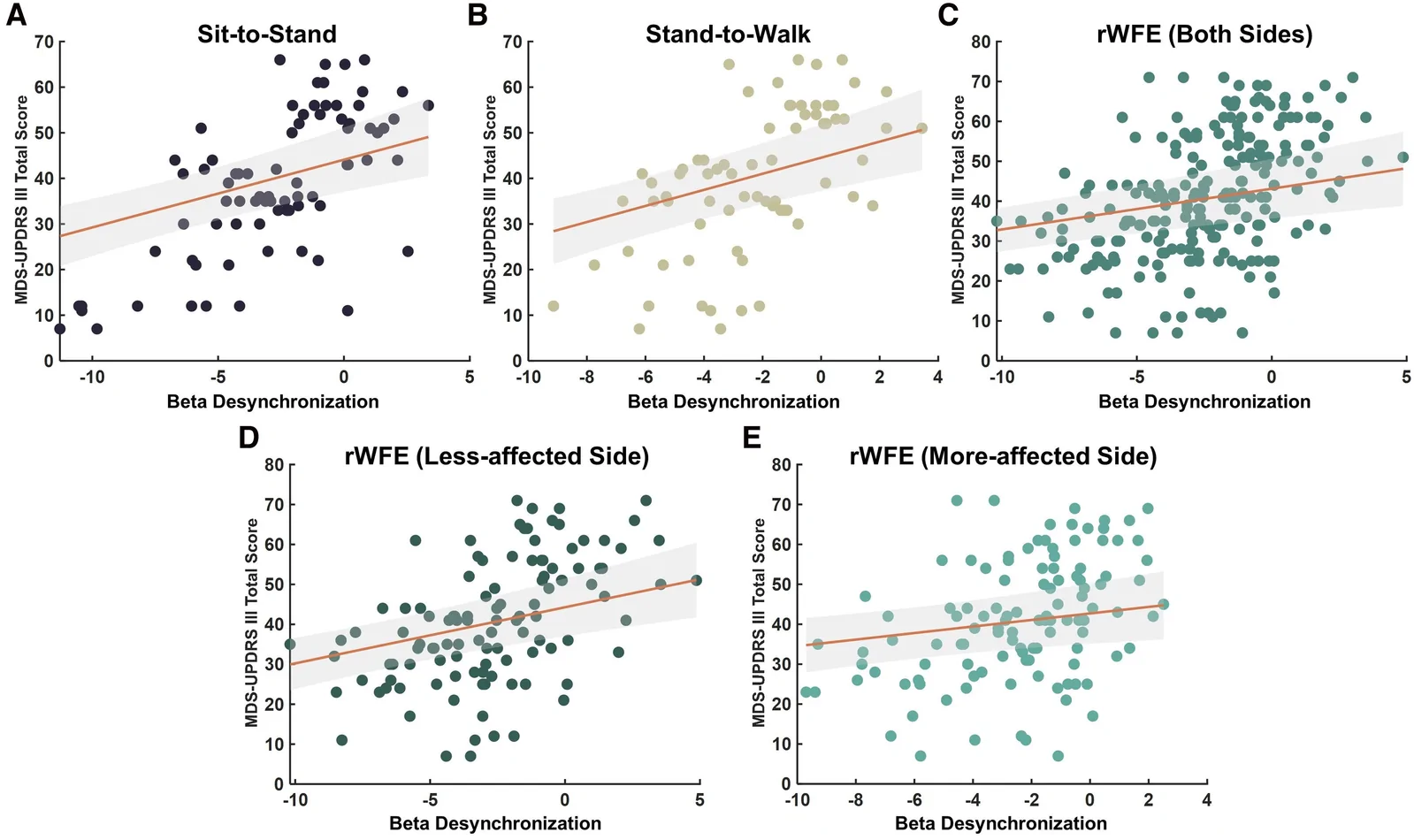
**Association between pre-movement initiation subthalamic nucleus (STN) beta desynchronization and Movement Disorder Society-Unified Parkinson’s Disease Rating Scale part III (MDS-UPDRS III) total score for different motor tasks.** (**A**) Sit-to-stand. (**B**) Stand-to-walk. (**C**) Repetitive wrist flexion and extension (rWFE) of both wrists. (**D**) rWFE of less-affected (LA) side only. (**E**) rWFE of more-affected (MA) side only. Beta desynchronization is defined as beta power during pre-movement initiation normalized to resting beta power; negative values indicate greater desynchronization. Each dot represents a single measurement per participant and visit. For beta desynchronization, values from each STN are plotted separately. The red line shows the average estimated slope of the association between beta power and MDS-UPDRS III total score, and the grey region represents the 95% confidence interval for the linear mixed-effects model (LMEM). (LMEM; sit-to-stand, *n* = 78, *β* = 1.5 [0.83, 2.1], *t* = 4.5, *P* < 0.001; stand-to-walk, *n* = 70, *β* = 1.8 [1.0, 2.5], *t* = 4.7, *P* < 0.001; rWFE, *n* = 228, *β* = 1.0 [0.53, 1.5], *t* = 4.1, *P* < 0.001; rWFE_LA_, *n* = 114, *β* = 1.4 [0.76, 2.0], *t* = 4.3, *P* < 0.001; rWFE_MA_, *n* = 114, *β* = 0.82 [0.16, 1.5], *t* = 2.5, *P* = 0.015).

LMEM analyses revealed a significant negative relationship between the degree of pMI STN beta desynchronization and motor impairment severity, as measured by the MDS-UPDRS III total score. Specifically, less pMI beta desynchronization (smaller reductions in beta power) was associated with greater motor impairment across all tasks (LMEM; sit-to-stand, *β* = 1.5 [0.83, 2.1], *t* = 4.5, *P* < 0.001; stand-to-walk, *β* = 1.8 [1.0, 2.5], *t* = 4.7, *P* < 0.001; rWFE, *β* = 1.0 [0.53, 1.5], *t* = 4.1, *P* < 0.001; Fig. 3A–C). This relationship was task-general, as evidenced by the lack of significant interaction effects between task and beta desynchronization (LMEM; sit-to-stand versus stand-to-walk, *β* = 0.39 [−0.64, 1.4], *t* = 0.74, *P* = 0.46; sit-to-stand versus rWFE, *β* = −0.43 [−1.25, 0.38], *t* = −1.1, *P* = 0.30). When comparing the beta desynchronization in the STNs contralateral to the MA and LA sides during the rWFE task, the LA side showed a relatively stronger association with total motor impairment (LMEM; rWFE_LA_, *β* = 1.4 [0.76, 2.0], *t* = 4.3, *P* < 0.001; rWFE_MA_, *β* = 0.82 [0.16, 1.5], *t* = 2.5, *P* = 0.015; Fig. 3D and E). Direct statistical comparison confirmed that the difference in association strength between the LA and MA sides was not statistically significant (*P* = 0.20).

**Figure 4:**
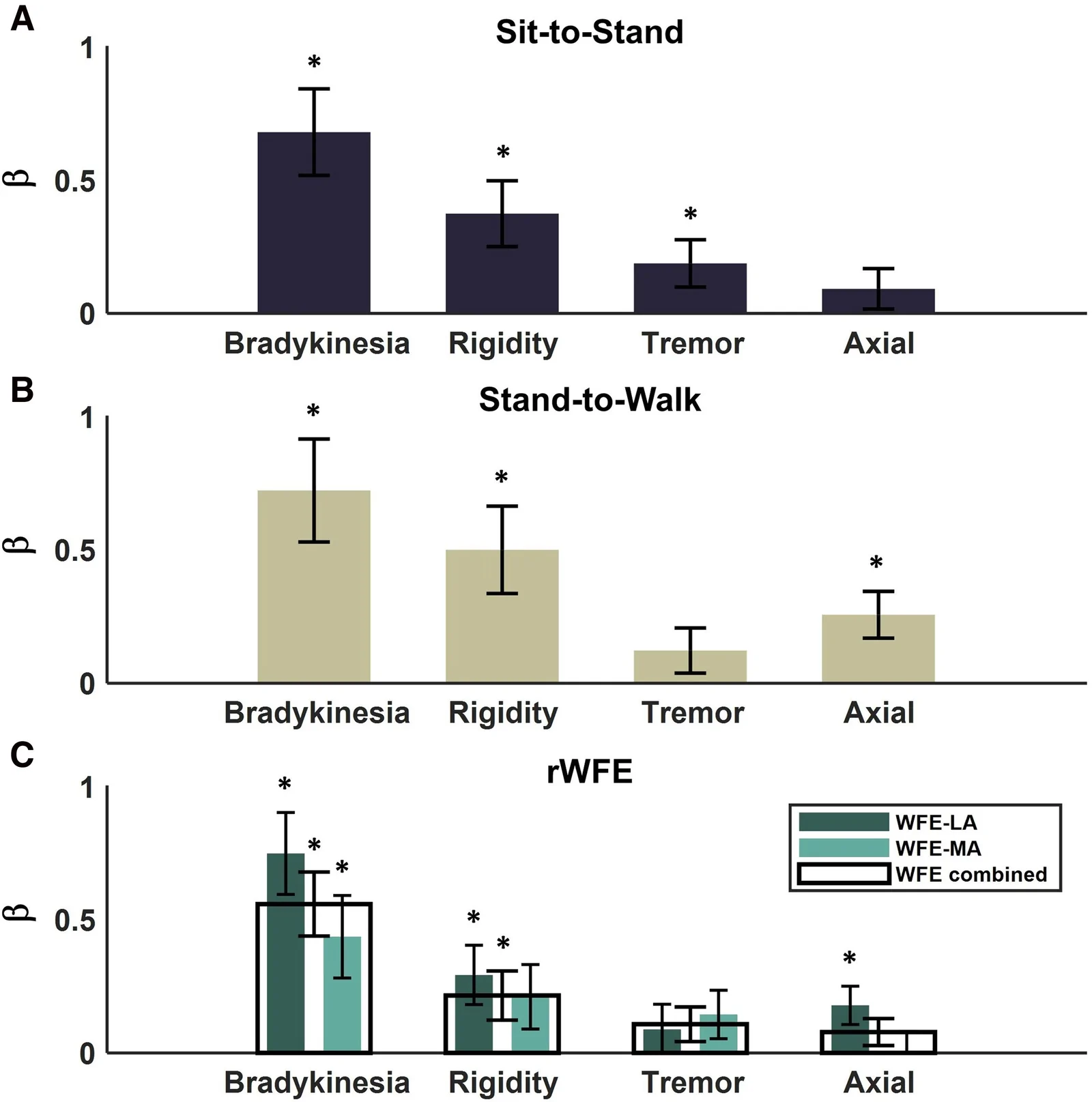
**Fixed effect coefficient (β) of the linear mixed-effects model (LMEM) for pre-movement initiation beta desynchronization and Movement Disorder Society-Unified Parkinson’s Disease Rating Scale part III (MDS-UPDRS III) subscores.** (**A**) Sit-to-stand. (**B**) Stand-to-walk. (**C**) Repetitive wrist flexion and extension (rWFE) of less-affected (LA) side, more-affected (MA) side and both sides (*β* ± SE; *, LMEM, *P* < 0.05). The selected categories of subscore include bradykinesia, rigidity, tremor and axial. Sit-to-stand (*n* = 14, LMEM; bradykinesia, *β* = 0.68 [0.36, 1.0], *t* = 4.2, *P* < 0.001; rigidity, *β* = 0.37 [0.13, 0.62], *t* = 3.0, *P* = 0.0034; tremor, *β* = 0.19 [0.011, 0.36], *t* = 2.1, *P* = 0.038; axial, *β* = 0.092 [−0.059, 0.24], *t* = 1.2, *P* = 0.23). Stand-to-walk (*n* = 14, LMEM; bradykinesia, *β* = 0.72 [0.34, 1.1], *t* = 3.7, *P* < 0.001; rigidity, *β* = 0.50 [0.17, 0.83], *t* = 3.1, *P* = 0.0033; tremor, *β* = 0.12 [−0.047, 0.29], *t* = 1.4, *P* = 0.15; axial, *β* = 0.26 [0.082, 0.43], *t* = 2.9, P = 0.0047). rWFE-combined (*n* = 15, LMEM; bradykinesia, *β* = 0.56 [0.32, 0.80], *t* = 4.7, *P* < 0.001; rigidity, *β* = 0.22 [0.033, 0.40], *t* = 2.3, *P* = 0.020; tremor, *β* = 0.11 [−0.020, 0.24], *t* = 1.7, *P* = 0.099; axial, *β* = 0.079 [−0.021, 0.18], *t* = 1.5, *P* = 0.12). rWFE_LA_ (*n* = 15, LMEM; bradykinesia, *β* = 0.75 [0.44, 1.1], *t* = 4.9, *P* < 0.001; rigidity, *β* = 0.29 [0.072, 0.51], *t* = 2.6, *P* = 0.0098; tremor, *β* = 0.089 [−0.098, 0.28], *t* = 0.94, *P* = 0.35; axial, *β* = 0.18 [0.036, 0.32], *t* = 2.5, *P* = 0.014). rWFE_MA_ (*n* = 15, LMEM; bradykinesia, *β* = 0.44 [0.13, 0.74], *t* = 2.8, *P* = 0.0057; rigidity, *β* = 0.21 [−0.029, 0.45], *t* = 1.7, *P* = 0.084; tremor, *β* = 0.14 [−0.036, 0.32], *t* = 1.6, *P* = 0.11; axial, *β* = 0.006 [−0.14, 0.15], *t* = 0.08, *P* = 0.94).

When examining associations between pMI beta desynchronization and MDS-UPDRS III subscores for the sit-to-stand task, significant negative relationships were observed for bradykinesia (LMEM; *β* = 0.68 [0.36, 1.0], *t* = 4.2, *P* < 0.001), rigidity (*β* = 0.37 [0.13, 0.62], *t* = 3.0, *P* = 0.0034) and tremor (*β* = 0.19 [0.011, 0.36], *t* = 2.1, *P* = 0.038), but not for axial symptoms (*β* = 0.092 [−0.059, 0.24], *t* = 1.2, *P* = 0.23; Fig. 4A and B). Direct statistical comparison confirmed that bradykinesia (slope = 0.60) had a significantly stronger association with reduced pMI beta desynchronization compared to axial symptoms (slope = 0.20, *P* = 0.024).

For the stand-to-walk task, significant associations with reduced pMI beta desynchronization were found for bradykinesia (LMEM; *β* = 0.72 [0.34, 1.1], *t* = 3.7, *P* < 0.001), rigidity (*β* = 0.50 [0.17, 0.83], *t* = 3.1, *P* = 0.0033) and axial symptoms (*β* = 0.26 [0.082, 0.43], *t* = 2.9, *P* = 0.0047), while tremor was not significant (*β* = 0.12 [−0.047, 0.29], *t* = 1.4, *P* = 0.15; Fig. 4B). Direct comparisons indicated that bradykinesia (slope = 0.66) had a significantly stronger association with reduced pMI beta desynchronization compared to tremor (slope = 0.18, *P* = 0.022).

For the rWFE task (both sides combined), significant associations with reduced pMI beta desynchronization were observed only for bradykinesia (LMEM; *β* = 0.56 [0.32, 0.80], *t* = 4.7, *P* < 0.001) and rigidity (*β* = 0.22 [0.034, 0.40], *t* = 2.3, *P* = 0.020; Fig. 4C). Bradykinesia (slope = 0.56) showed significantly stronger associations compared to rigidity (slope = 0.22, *P* = 0.0055), tremor (slope = 0.12, *P* < 0.001) and axial symptoms (slope = 0.084, *P* < 0.001).

When analysing the rWFE task separately by MA and LA sides, bradykinesia showed significant associations for both sides (LMEM; MA, *β* = 0.44 [0.13, 0.74], *t* = 2.8, *P* = 0.0057; LA, *β* = 0.75 [0.44, 1.1], *t* = 4.9, *P* < 0.001; difference between slopes: *P* = 0.18; Fig. 4C). Rigidity (*β* = 0.29 [0.072, 0.51], *t* = 2.6, *P* = 0.0098) and axial symptoms (*β* = 0.18 [0.036, 0.32], *t* = 2.5, *P* = 0.014) were significant only for the LA side. Bradykinesia showed statistically stronger associations compared to other subscores: for the LA side, bradykinesia (slope = 0.76) was significantly stronger than rigidity (slope = 0.29, *P* = 0.0034), axial symptoms (slope = 0.17, *P* < 0.001) and tremor (slope = 0.11, *P* < 0.001); for the MA side, bradykinesia (slope = 0.42) was significantly stronger than axial symptoms (slope = 0.012, *P* = 0.012).

### Association between pre-movement subthalamic beta desynchronization and acceleration index

**Figure 5:**
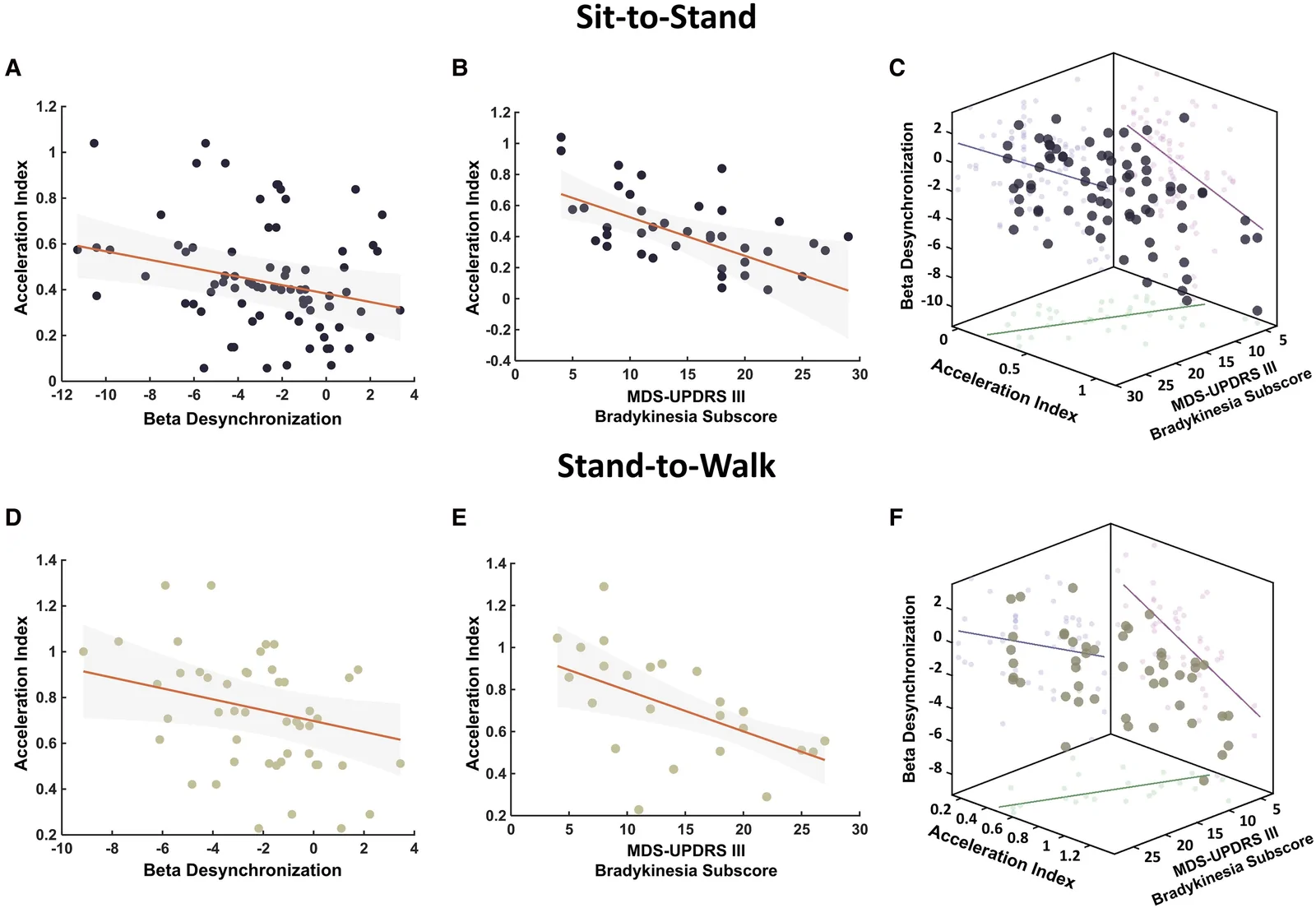
**Association between pre-movement initiation (pMI) beta desynchronization, acceleration index (AI) and Movement Disorder Society-Unified Parkinson’s Disease Rating Scale part III (MDS-UPDRS III) bradykinesia subscores for sit-to-stand (A–C) and stand-to-walk tasks (D–F).** (**A** and **D**) Beta desynchronization versus AI. (**B** and **E**) Bradykinesia subscore versus AI. (**C** and **F**) 3D summary plots. Beta desynchronization is defined as beta power during pMI normalized to resting beta power; negative values indicate greater desynchronization. Each dot represents a single measurement per participant and visit. For beta desynchronization, values from each subthalamic nucleus (STN) are plotted separately. Red lines indicate fitted slopes from linear mixed-effects models (LMEM); grey shaded regions represent 95% confidence intervals. For stand-to-walk, trials in which participants failed to execute a distinct initial step were excluded from the analysis. pMI beta desynchronization and AI (LMEM; sit-to-stand, *n* = 78, *β* = −0.018 [−0.033, −0.0044], *t* = −2.6, *P* = 0.011; stand-to-walk, *n* = 48, *β* = −0.024 [−0.047, −0.00082], *t* = −2.1, *P* = 0.043). AI and MDS-UPDRS III bradykinesia subscores (LMEM; sit-to-stand, *n* = 39, *β* = −0.025 [−0.042, −0.0082], *t* = −3.0, *P* = 0.0040; stand-to-walk, *n* = 24, *β* = −0.019 [−0.031, −0.0077], *t* = −3.3, *P* = 0.0017).

Further LMEM analyses demonstrated that greater pMI beta desynchronization was significantly associated with higher AI for both sit-to-stand (*β* = −0.018 [−0.033, −0.0044], *t* = −2.6, *P* = 0.011; Fig. 5A) and stand-to-walk (*β* = −0.024 [−0.047, −0.00082], *t* = −2.1, *P* = 0.043; Fig. 5D). Additionally, higher AIs were significantly associated with lower MDS-UPDRS III bradykinesia subscores for both tasks (sit-to-stand: *β* = −0.025 [−0.042, −0.0082], *t* = −3.0, *P* = 0.0040; stand-to-walk: *β* = −0.019 [−0.031, −0.0077], *t* = −3.3, *P* = 0.0017; Fig. 5B and E). These findings indicate that greater beta desynchronization during movement preparation is linked to more vigorous movement initiation and less severe bradykinesia (Fig. 5C and F).

## Discussion

In this study, we characterized beta-band oscillatory activity in the STN during the pMI phase across multiple motor tasks in individuals with Parkinson’s disease. There was consistent beta desynchronization in the STN during movement preparation across all motor tasks. For the first time, we demonstrated that reduced beta desynchronization during pMI was significantly associated with greater overall motor impairment, particularly bradykinesia and rigidity. Furthermore, reduced beta desynchronization was associated with less vigorous movement initiation, as indicated by lower acceleration indices. Taken together, these findings provide novel evidence that impaired subcortical beta modulation during movement preparation may contribute to motor dysfunction in Parkinson’s disease, highlighting the potential of pMI STN beta activity as a biomarker for the severity of motor impairment.

### Pre-movement subthalamic beta desynchronization is a robust feature across motor tasks

Our findings extend previous studies that initially characterized pMI beta-band modulation in the STN. Kühn *et al*.^21^ demonstrated that beta desynchronization in the STN occurs prior to externally cued button-press movements, with the latency of this desynchronization correlating with reaction time. Importantly, they also observed that beta desynchronization was reduced when movements were inhibited, collectively suggesting that STN beta modulation plays a functional role in motor preparation and initiation.^21^ Similarly, Williams *et al*.^22^ showed that beta-band activity in the STN was modulated by predictive behavioural cues, indicating that beta desynchronization may reflect anticipatory motor processes and preparation for upcoming movements. In preliminary results from three individuals with Parkinson’s disease, Priori *et al*.^31^ reported that beta desynchronization in the STN and globus pallidus internus (GPi) began during the pMI phase of voluntary finger movements and reached maximal desynchronization during movement execution. They observed consistent pMI desynchronization in the high-beta frequency band (∼26 Hz) in both STN and GPi, whereas the low-beta band (∼18 Hz) showed more variable modulation across patients.^31^ Our study expands upon these foundational findings by demonstrating consistent beta desynchronization in the STN prior to movement initiation across three distinct motor tasks (sit-to-stand, stand-to-walk and rWFE). Importantly, we found no statistical difference in the degree of beta desynchronization across these tasks, suggesting that pMI beta desynchronization in the STN is a robust and generalizable neural feature of motor preparation, independent of the specific motor demands.

### Pre-movement subthalamic beta desynchronization reflects overall motor impairment severity

To our knowledge, the results of this study demonstrate for the first time a robust association between reduced pMI beta desynchronization in the STN and greater overall motor impairment severity across all three motor tasks. Previous studies have identified exaggerated beta power as a hallmark of motor impairment in Parkinson’s disease. For example, Neumann *et al*.^11^ reported that elevated resting beta power in the STN correlated with higher UPDRS III scores, suggesting impaired beta modulation as a neural correlate of motor deficits. Shreve *et al*.^13^ showed that beta power was greater in the MA STN compared to the LA STN. Little and Brown highlighted beta oscillations as antikinetic signals whose impaired suppression contributes to motor impairment.^10^ Our study expands these findings by specifically demonstrating that impaired beta modulation during the critical pMI phase is consistently associated with motor impairment severity across both upper-extremity and whole-body tasks.

### Pre-movement beta desynchronization’s selective link to bradykinesia, rigidity and movement acceleration

Across all tasks, bradykinesia and rigidity subscores demonstrated the relatively stronger associations with pMI beta desynchronization, with bradykinesia consistently exhibiting the numerically strongest relationship. This selective association is consistent with previous literature. For instance, Kühn *et al*.^14^ found that pathological beta synchronization in the STN specifically correlated with bradykinesia and rigidity, but not tremor, suggesting a selective neural mechanism underlying these cardinal symptoms. Ray *et al*.^17^ similarly reported that reductions in STN beta power following dopaminergic medication correlated specifically with improvements in bradykinesia. Our findings extend these observations by demonstrating that impaired beta modulation, even during movement preparation, specifically relates to bradykinesia and rigidity severity across multiple motor tasks. Furthermore, during tasks involving whole-body movement, we found significant associations with tremor in the sit-to-stand task and with axial symptoms in the stand-to-walk task.

Our kinematic analyses further reinforce this clinical relationship by demonstrating that greater pMI beta desynchronization is associated with higher acceleration indices reflecting more vigorous movement initiation during sit-to-stand and stand-to-walk tasks. Additionally, higher acceleration indices correlated with lower bradykinesia subscores, underscoring the functional link between impaired beta modulation, reduced movement acceleration and clinical bradykinesia. Previous studies have similarly connected impaired beta modulation to reduced movement acceleration and slower initiation. For instance, Lofredi *et al*.^32^ found that the percentage time spent in beta bursts correlated with slower velocities and lower acceleration during upper-limb movements, directly linking beta oscillations to bradykinetic motor behaviour. Likewise, Torrecillos *et al*.^33^ reported that greater beta suppression was associated with faster reaction times and more vigorous movements. Kühn *et al*.^21^ previously demonstrated a relationship between pMI beta desynchronization and reaction time during simple movements; however, our findings significantly expand upon this earlier work by showing that pMI beta desynchronization relates not only to reaction time but also to subsequent movement acceleration during complex, whole-body functional tasks. Thus, our results highlight a broader functional role of beta modulation deficits in Parkinson’s disease, extending beyond reaction time alone to encompass the quality and acceleration of movement initiation in clinically relevant motor behaviours.

### Neural mechanisms for the link between pre-movement beta desynchronization and motor impairment

The observed associations between impaired pMI beta desynchronization and clinical motor impairment, particularly bradykinesia and rigidity, may reflect underlying dysfunction in basal ganglia-thalamocortical motor circuits in movement preparation. While beta desynchronization is a broad ‘release’ signal observable even during non-motor tasks, its robust correlation with movement acceleration in our data highlights its specific role in gating motor output. Beta oscillations in the STN are thought to represent synchronized inhibitory activity within basal ganglia networks, which must be effectively suppressed (desynchronized) to facilitate movement initiation and execution.^10,12,34,35^ In Parkinson’s disease, exaggerated beta synchronization and impaired beta suppression may reflect pathological hyperactivity of the indirect pathway and reduced activity of the direct pathway.^36,37^ While recent evidence challenges the strict segregation of these pathways, suggesting a more complex interplay of co-activated populations,^38,39^ impaired beta desynchronization functionally represents excessive net inhibitory output from the basal ganglia to cortical motor areas. This excessive inhibition could manifest clinically as akinesia, bradykinesia and rigidity.^14,17^ Supporting this interpretation, animal and computational modelling studies have demonstrated that increased beta synchronization in basal ganglia circuits directly contributes to reduced cortical excitability and impaired motor initiation.^36,37^ Furthermore, therapeutic interventions such as dopaminergic medication and DBS, which effectively reduce pathological beta synchronization, consistently improve bradykinesia and rigidity symptoms.^9,17,40^ Thus, impaired pMI beta desynchronization observed in our study may represent a neural signature of disrupted basal ganglia-thalamocortical signalling, providing a mechanistic explanation for the observed clinical associations and highlighting its potential as a therapeutic target.

### Complexity of motor tasks influences the strength of beta desynchronization associations

The strength of the association between pMI beta desynchronization and motor impairment severity varied across tasks, with the strongest numerical association observed during the more complex stand-to-walk task, followed by sit-to-stand and then rWFE. This suggests that tasks involving greater complexity and whole-body coordination may more sensitively reflect underlying neural dysfunction in Parkinson’s disease. Previous work has demonstrated that complex tasks involving transitions (e.g. sit-to-stand and gait initiation) are more sensitive to detecting motor impairment severity than simple, isolated limb movements.^41^ Similarly, neural biomarkers of motor impairment have been observed to be more pronounced during complex whole-body tasks, including gait and posture transitions, compared to simpler tasks.^42^ From a physiological perspective, complex tasks require greater integration of sensory feedback, motor planning and coordination across multiple muscle groups and neural circuits to initiate, potentially amplifying the impact of impaired pMI beta modulation in Parkinson’s disease. Clinically, this underscores the importance of incorporating complex, functionally relevant tasks into patient assessments, as these tasks may better capture subtle deficits in neural control and coordination that simpler tasks might overlook.

### Asymmetry in beta desynchronization associations between more- and less-affected sides

Interestingly, pMI beta desynchronization showed numerically stronger associations with motor impairment severity on the LA side than on the MA side during the rWFE task. This distinction suggests that the LA side may serve as a more sensitive gauge of global motor impairment, whereas the more-affected network may be pathologically saturated, potentially introducing noise that obscures the linear relationship. Our observation aligns with previous work suggesting the LA side may be more sensitive to clinical changes. For instance, our previous kinematic study^43^ showed that the velocity of wrist pronation-supination on the LA side declined at a significantly steeper rate with increasing disease severity compared to the MA side. This concept is further supported by work from Heinrichs-Graham *et al*.,^44^ who found that the degree of symptom laterality in Parkinson’s disease is directly reflected by the asymmetry of beta-band activity during movement. Structural asymmetry, such as reduced transcallosal connectivity,^45^ may further contribute to functional differences in neural modulation capacity and explain heightened sensitivity on the LA side. Therefore, our results reinforce the value of bilateral assessment and highlight the LA side as a potentially more sensitive marker for detecting subtle neural dysfunction in Parkinson’s disease.

### Limitations and potential future directions

Several limitations of our study should be acknowledged. First, our sample size was relatively small (*n* = 16), potentially limiting statistical power and generalizability. Second, medication and DBS washout conditions, while necessary for experimental control, may limit direct translation to clinical settings. Finally, our analyses focused on a limited number of trials and visits, as well as a relatively short pMI analysis window. While the 0.25-s sliding window for beta power analysis provided the temporal resolution needed for this brief phase, it limits finer frequency analyses (e.g. distinguishing low versus high beta). Furthermore, we did not assess discrete beta burst metrics (e.g. duration, rate) due to the methodological challenges of defining robust burst thresholds within the short, non-stationary pMI window. Therefore, future work is required to explore the specific temporal dynamics underlying reduced desynchronization, such as the potential persistence of transient beta bursts. Additionally, as the pMI window was time-locked to movement in a cued task, potential contributions from cue-evoked processes cannot be fully excluded. Future studies with larger samples, longitudinal designs and broader task batteries with jittered cue-response intervals are needed to confirm and extend our findings.

Future research directions include longitudinal studies assessing changes in pMI beta modulation over disease progression, investigations of the effects of dopaminergic medication and DBS on pMI beta modulation and studies exploring cortical-subcortical interactions during movement preparation using simultaneous cortical and STN recordings. Additionally, our findings may inform the development of adaptive DBS strategies that leverage pMI beta-band dynamics to optimize therapeutic outcomes.^19,20,46,47^

In conclusion, our findings demonstrate that greater beta desynchronization during movement preparation is closely associated with more vigorous movement initiation and less severe motor impairment in Parkinson’s disease, particularly bradykinesia. These results highlight the potential of pMI STN beta desynchronization as a sensitive biomarker for motor impairment and underscore the importance of characterizing neural dynamics during movement preparation to better understand motor dysfunction in Parkinson’s disease.

## Supplementary Material

fcag245_Supplementary_Data

## Data Availability

The data supporting the findings of this study are available from the corresponding author upon reasonable request. Analysis codes are available at the following GitHub repository: https://github.com/slowriver322/pMI_BetaDesync_BrainCom.git
