# Supplementary material for: Reduced pre-movement subthalamic beta desynchronization marks motor deficit in Parkinson’s disease
Source: Brain Commun. 2026 Jun 25;8(4):fcag245. doi: 10.1093/braincomms/fcag245 (PMC13343376; doi:10.1093/braincomms/fcag245)
Supplement: fcag245_Supplementary_Data [file fcag245_supplementary_data.docx]

**Supplementary Table 1. Montreal Neurological Institute (MNI) coordinates of** **subthalamic nucleus (STN) deep brain stimulation lead localization**

| **ID** | **STN** | **Center Contact of Recording Pair** | **X** | **Y** | **Z** |
| --- | --- | --- | --- | --- | --- |
| **1** | Left | 1 | -13.40 | -13.97 | -5.88 |
|  | Right | 9 | 11.73 | -13.11 | -6.70 |
| **2** | Left | 2 | -14.21 | -13.36 | -4.42 |
|  | Right | 9 | 11.21 | -13.23 | -7.76 |
| **3** | Left | 2 | -11.40 | -11.53 | -5.31 |
|  | Right | 10 | 10.85 | -11.28 | -5.72 |
| **4** | Left | 1 | -14.59 | -13.02 | -5.62 |
|  | Right | 9 | 12.67 | -13.78 | -5.29 |
| **5** | Left | 1 | -11.78 | -14.71 | -6.97 |
|  | Right | 9 | 11.59 | -12.30 | -6.76 |
| **6** | Left | 2 | -12.24 | -13.91 | -3.52 |
|  | Right | 10 | 11.23 | -12.09 | -6.27 |
| **7** | Left | 1 | -12.60 | -14.62 | -6.24 |
|  | Right | 10 | 11.10 | -13.67 | -6.98 |
| **8** | Left | 1 | -12.41 | -13.35 | -5.45 |
|  | Right | 10 | 10.37 | -13.58 | -7.81 |
| **9** | Left | 1 | -11.29 | -14.60 | -7.11 |
|  | Right | 10 | 12.21 | -13.58 | -5.87 |
| **10** | Left | 2 | -13.11 | -13.80 | -5.58 |
|  | Right | 10 | 11.83 | -12.68 | -6.15 |
| **11** | Left | 1 | -12.36 | -14.07 | -4.33 |
|  | Right | 9 | 11.70 | -11.97 | -4.72 |
| **12** | Left | 1 | -13.22 | -14.41 | -6.99 |
|  | Right | 10 | 15.65 | -11.63 | -3.98 |
| **13** | Left | 1 | -12.46 | -14.11 | -6.72 |
|  | Right | 9 | 10.97 | -12.86 | -5.97 |
| **14** | Left | 2 | -13.03 | -13.69 | -5.21 |
|  | Right | 9 | 10.76 | -13.75 | -7.58 |
| **15** | Left | 1 | -12.72 | -13.00 | -5.28 |
|  | Right | 9 | 11.94 | -11.77 | -4.52 |
| **16** | Left | 1 | -13.67 | -13.37 | -6.33 |
|  | Right | 9 | 12.26 | -12.16 | -5.06 |

X: Right (+) / Left (-); Y: Anterior (+) / Posterior (-); Z: Superior (+) / Inferior (-)

**Supplementary Table 2. Subthalamic nucleus (STN) deep brain stimulation electrode contact pairs and impedance measured at each visit month after implantation**

| **ID** | **STN** | **Contact Pair** | **Impedance (Ω)** | | | | |
| --- | --- | --- | --- | --- | --- | --- | --- |
|  |  |  | **1 Month** | **3 Month** | **6 Month** | **9 Month** | **12 Month** |
| 1 | Left | 0-2 | 4677 | 5808 | 7074 | 7008 | 6754 |
|  | Right | 8-10 | 4008 | 4924 | 5590 | 5995 | 1616 |
| 2 | Left | 1-3 | 1710 | 3694 | 3761 | 3801 | 3654 |
|  | Right | 8-10 | 4811 | 5231 | 5512 | 5471 | 4654 |
| 3 | Left | 1-3 | 4571 | 5134 | 2530 | 1968 | 1877 |
|  | Right | 9-11 | 4200 | 6049 | 6292 | 5721 | 4176 |
| 4 | Left | 0-2 | 4702 | 4321 | 5378 | 4823 | 5620 |
|  | Right | 8-10 | 4130 | 4040 | 3663 | 3192 | 3040 |
| 5 | Left | 0-2 | 3522 | 4039 | 4465 | - | - |
|  | Right | 8-10 | 3259 | 3230 | 3774 | - | - |
| 6 | Left | 1-3 | 5445 | 4306 | 5266 | 5740 | 3918 |
|  | Right | 9-11 | 4717 | 5073 | 5445 | 5405 | 3857 |
| 7 | Left | 0-2 | 4604 | 4944 | 3806 | 3766 | 3886 |
|  | Right | 9-11 | 4150 | 4283 | 4771 | 5032 | 4621 |
| 8 | Left | 0-2 | 4724 | 4489 | 2715 | - | 2438 |
|  | Right | 9-11 | 3456 | 3375 | 2906 | - | 2405 |
| 9 | Left | 0-2 | 3198 | 3562 | - | 5283 | - |
|  | Right | 9-11 | 3553 | 4367 | - | 4407 | - |
| 10 | Left | 1-3 | - | 4177 | - | - | 4131 |
|  | Right | 9-11 | - | 4154 | - | - | 4177 |
| 11 | Left | 0-2 | 5082 | 6371 | 6661 | - | 6020 |
|  | Right | 8-10 | 5382 | 4737 | 5705 | - | 5154 |
| 12 | Left | 0-2 | - | 3928 | - | 3020 | - |
|  | Right | 9-11 | - | 4298 | - | 4402 | - |
| 13 | Left | 0-2 | 3325 | 4025 | 4021 | 4492 | 4612 |
|  | Right | 8-10 | 4471 | 4732 | 2450 | 1676 | 1692 |
| 14 | Left | 1-3 | 4563 | 5032 | 6276 | 7056 | 5426 |
|  | Right | 8-10 | 4347 | 4771 | 5482 | 4950 | 5053 |
| 15 | Left | 0-2 | 2564 | - | 3322 | 4165 | 1661 |
|  | Right | 8-10 | 5176 | - | 4003 | 4165 | 3875 |
| 16 | Left | 0-2 | 5500 | - | 7132 | - | 7132 |
|  | Right | 8-10 | 5755 | - | 5366 | - | 7416 |
